# 

**Published:** 1892-04-16

**Authors:** 


**Notices of Births, Deaths, and Marriages,** are inserted in this column at a charge of 2s. 6d. for an announcement not exceeding four lines.

### NOTICE TO CORRESPONDENTS.

All MS., Letters, Books for Review, and other matters intended for the Editor should be addressed The Editor, The Lodge, Porchester Square, London, W.

The Editor cannot undertake to return rejected M.S., even when accompanied by stamped directed envelope.

**Notice to Advertisers and Subscribers.**

All Advertisements, Orders for Copies of The Hospital, and Business Communications should be addressed to The Publisher, *not to the Editor*, The Hospital, 140, Strand, W.C.

The Cost of Subscription, post free, is as follows:—Per week, 2½d.; per quarter, 2s. 6d.; per half-year, 5s.; per annum, 8s. 6d. Foreign:—Per annum, 10s. 8d.; payable, in all cases, in advance.

**"Burdett's Hospital Annual," 1891—1892.**

Third year of publication. 600 pp. Boards, gilt lettering. A most complete guide to British and Colonial Hospitals, Dispensaries, Nursing and Convalescent Institutions. and Asylums. Price 3s. 6d. Published by The Scientific Press (Limited), 140, Strand, W.C.

**NOTICE.—The Index for Vol. X. (ending September, 1891), is on sale at the Office of this Journal, price 2½d., post free.**

### SCRAPS AND GLEANINGS.

DURING the past year sixty-two patients were admitted to the Falkirk Cottage Hospital.

IT is understood that Dr. Thorne Thorne will be appointed Medical Officer to the Local Government Board.

THE sum of about £100 was last week discovered at Sittingbourne whilst unloading a barge of rubbish from London.

MR. EDWARD LAWSON, of the *Daily Telegraph*, president of the Institute of Journalists, has given a donation of £200 to the orphan fund of the institute.

DURING the last fortnight upwards of 73 tons of flowers have arrived at Penzance from the Scilly Islands on their way to the London and Birmingham markets.

A SERIOUS epidemic of influenza exists among horses in London, and continues to spread. It is stated that the outbreak is more virulent than any which has preceded it.

THE Ameer of Afghanistan enclosed his missive of condolence to the Queen on the death of the Duke of Clarence and Avondale in a casket of sterling gold, about a pound in weight.

HER MAJESTY has been pleased to confer the honour of knighthood upon Dr. George Buchanan, on his retirement from the post of Medical Officer to the Local Government Board.

THE Earl of Strafford will take the chair at a festival dinner in aid of the City of London Hospital for Diseases of the Chest, at the Whitehall Rooms, Hotel Metropole, on Tuesday, May 24th.

THE Prince and Princess of Wales will lay the foundation-stone of the new wing to St. Mary's Hospital in October, and have given permission for it to be named the "Clarence Memorial Wing."

THE Committee of the Grimsby and District Hospital have disposed of a large number of hospital collection boxes for use on fishing vessels, and it is hoped that the result will be most beneficial to the funds of the hospital.

AN epidemic of scarlet fever has broken out in the neighbourhood of Bexley Heath. It was first noticed among members of families whose milk was supplied from London. The national and private schools of the locality are closed for one month.

THE Queen has given orders for the appointment of Surgeon-Captain George Ernest Hale, Medical Staff, to be a Companion of the Distinguished Service Order, in recognition of his services during the operations against the Kachins, and in the Wuntho District, Upper Burmah.

THE Queen has been pleased to approve the appointment of Mr. J. A. Froude to be Regius Professor of Modern History, in the University of Oxford in the room of the late Professor Freeman. Mr. Froude was educated at Westminster and Oriel College, Oxford, and is an honorary Fellow of Exeter.

THE Committee of the Great Northern Central Hospital, Holloway Road, have decided to erect the additional wing, which will complete the new buildings, as a memorial to the late Duke of Clarence. The cost of building, with the necessary furniture and fittings, will amount to about £30,000.

DURING the quarter ending March 31st of the present year 286 in-patients were admitted to the Devonshire Hospital, and Buxton Bath Charity, Buxton. Of these 191 were discharged as "improved," 5 as no better, 1 died of pneumonia in the hospital, and 89 remained on the books at the end of the quarter.

A GARDEN party given by Lady Havelock, at Kandy, last month, was brought to an abrupt conclusion by the arrival of a swarm of bees, which mercilessly stung guests, carriage horses, and drivers so severely that a scene of indescribable confusion followed. The visitors quickly dispersed, leaving the bees masters of the situation.

THE twenty-sixth festival of the Royal Medical Benevolent College, Epsom, was held recently at the Hotel Metropole, the Lord Mayor in the chair. A gift of £1,000 from the proprietors of the *Lancet* was announced as part of the subscription list towards the enlargement scheme, entailing an outlay of £26,000, which is being promoted.

THE Royal Hospital for Children and Women, Waterloo Bridge Road, S.E., situated in a very poor neighbourhood, appeals for the sum of £1,500 to clear the deficiency of the past year. The hospital is quite full, but some of the wards must be closed unless the amount asked for is quickly realised. During 1891 7,000 out-patients, and nearly 600 in-patients were admitted.

THE Queen has most generously endowed four additional beds in the Hospital of the Infant Asylum at Hyères. According to her expressed wish they are respectively to bear the names of "Queen Victoria," "Princess Beatrice," "Duchess of Connaught," and "Princess Margaret Victoria of Connaught." Her Majesty's benefaction, it is stated, is deeply appreciated by the people of Hyères.

FOR the past year the receipts of the Stockport Infirmary amounted to £3,684 12s. 9½d., against an expenditure of £3,623 3s. 10d., leaving a balance to be carried forward of £58 0s. 9d. It was suggested at the annual meeting that the workpeople should be called upon to subscribe more liberally.

THE Moseley Convalescent Home for Children was opened on Saturday afternoon, April 2nd. The Mayor, after having, on behalf of the inhabitants of Birmingham and the districts, thanked Mr. and Mrs. Cadbury for their most generous gift, stated that in the building they had the space for 150 beds. The Committee propose to start the home with 50 beds, and they hope the public will quickly help them to provide the remaining 100.

A MEETING of the Institute of Civil Engineers of Ireland was held on the 6th inst., in their new hall, Dawson Street, Dublin. A paper was read by Mr. W. Kaye Parry, on "The Sewage Disposal of Isolated Dwellings." The author described and illustrated with diagrams several drainage and sewage-disposal works which he had carried out, including those at the workhouse at Loughlinstown, co. Dublin, for the Guardians of the Rathdown Union; at "Oaxton," Maywood, co. Kildare, for his grace the Duke of Leinster; and at many other places.

### NOTICE.

### FURNITURE AND APPLIANCES.

We purpose giving a descriptive account with illustrations of all the new appliances and furniture used in hospitals and asylums, and we desire to secure the co-operation and the superintendence of the medical staff, secretaries, and matrons, so that the whole ground may be covered by this section, which should prove very interesting and helpful in many ways. Each institution which has any fittings or furniture which are considered specially suitable or novel are invited to send a description with sketch for publication in this column. All communications should be marked "Furniture and Appliances," and addressed to the Editor of The Hospital, 140, Strand, W.C.

### THE STUDENT OF MEDICINE.

[All communications for this Supplement should be addressed to The Editor of The Hospital, at 140, Strand, with the words, "Students' Column," written in the left hand top corner of the envelope.]

### APPOINTMENTS.

Beesley, A. J., L.R.C.P.Lond., M.R.C.S., appointed Medical Officer of the West Derby Union Infirmary. Bennett, Vivian B., M.B., appointed Assistant Medical Officer to the Walton Workhouse Hospital. Boyd, Campbell, L.R.C.P., L.R.C.S.Irel., appointed Medical Officer to the Camberwell Provident Dispensary. Burnet, R. W., M.D., appointed Honorary Consulting Physician to the Royal Caledonian Asylum, Holloway, vice David W. Finlay, M.D., resigned. Cheyne, W. Watson, M.B., appointed Honorary Consulting Surgeon to the Royal Caledonian Asylum, Holloway, vice John Wood, F.R.C.S., deceased. Cotterell, Edward, F.R.C.S.Eng., appointed Surgeon to the Cancer Hospital, and Surgeon to the West End Hospital for Diseases of the Nervous System. Cruise, J. E. W., M.B., B.Ch.Irel., appointed Resident Medical Officer of the Grafton Street Hospital, Liverpool. Davies, Edward, M.D.St.And., M.R.C.S.Eng., appointed Deputy Coroner for the Eastern Division of Denbighshire. Elliot, E. A. S., L.R.C.P.Edin., M.R.C.S.Eng., appointed Medical Officer of the Workhouse of the Kingsbridge Union, vice John Elliot, M.R.C.S.Eng., resigned. Elliott, R. H., M.B., B.Ch.Irel., appointed Resident Medical Officer of the Netherfield Road Hospital, Liverpool. Glover, L. G., M.B., B.C.Cantab., appointed House-Physician to the Royal Hospital for Diseases of the Chest, City Road. Mulvany, John, M.R.C.S.Eng., L.R.C.P.Lond., appointed House-Surgeon to the Cottage Hospital, Walsall. Richards, J. S., M.D., B.S., appointed Resident Medical Officer to the Royal Hospital for Children and Women, Waterloo Bridge Road, vice J. M. Thorne, M.R.C.S.Eng., resigned. Roberts, John S.H., L.R.C.P.Lond., M.R.C.S.Eng., appointed Outdoor-Surgeon to the Swansea Hospital. Sinclair, Walter W., M.B., C.M.Aberd., appointed Junior House-Surgeon to the Birmingham and Midland Eye Hospital. Trend, T. Wm., M.D.St.And., M.R.C.P.Edin., M.R.C.S.Eng., appointed Physician to the Royal South Hants Infirmary. Wallace, William, M.A., L.D.S., appointed Lecturer on Dental Anatomy and Physiology at the Glasgow Dental Hospital, vice J. C. Woodburn, M.D.Glas., resigned. Wallis, F. C., M.B., B.S.Cantab., M.R.C.S., appointed Assistant Surgeon to the Metropolitan Hospital. Willett, Edgar, M.B., F.R.C.S., appointed Assistant Surgeon to the Metropolitan Hospital, Kingsland Road. The Queen has conferred the honour of knighthood upon Mr. George Buchanan, M.D., on his retirement from the post of Medical Officer to the Local Government Board.

### VACANCIES.

The following vacancies are announced this week, the amount of salary in each case being given after the name of the institution:

Birmingham City Asylum, Ruberry Hill, Bromsgrove, Clinical Assistant—Board, &c.

Bournemouth Friendly Societies' Medical Association, Junior Medical Officer

Cheshire County Asylum, Macclesfield, Senior Assistant Medical Officer, unmarried, and not under 26 years of age—Salary £150 per annum, with board, &c.

College of State Medicine, Research Scholarship, tenable for one year—Salary £100.

Cumberland Infirmary, Carlisle, House-Surgeon—Salary £70 per annum, with board, &c.

Haverstock Hill and Malden Road Provident Dispensary, 132, Malden Road, N.W., Medical Officer.

Lewes Dispensary and Infirmary and Victoria Hospital, Resident Medical Officer, doubly qualified—Salary £100 per annum, &c.

Manchester Royal Infirmary, Dispensary, and Lunatic Hospital or Asylum, Honorary Assistant-Physician.

Nottingham Borough Asylum, Mapperley Hill, Nottingham, Assistant Medical Officer, unmarried—Salary, £125 per annum, &c.

Paddington Green Children's Hospital, W., House-Surgeon. Appointment for six months—Salary at the rate of £55 5s. per annum, with Board, &c.

Parish of Glenelg, Inverness-shire, N.B., Medical Officer for the Southern Division—Salary, from Parochial Board, £50 a-year, free house and garden, and additional fixed salary of £100 from other sources.

### THE STUDENT OF MEDICINE—(*continued*).

Salop Infirmary, Shrewsbury, Dispenser—Salary £100 per annum, without residence or any extras.

Seamen's Hospital Society, Greenwich, S.E., Visiting Physician for Branch Hospital in the Royal Victoria and Albert Docks.

Seamen's Hospital Society, Greenwich, S.E., Visiting Ophthalmic Surgeon.

Victoria Hospital for Children, Queen's Road, Chelsea, Honorary Medical Officer to New Convalescent Branch at Broadstairs, to be opened in May.

### NOTES FROM THE SCHOOLS.

#### Guy's Hospital.

The current issue of the "Gazette" contains the notes of a clinical lecture on Typhoid Fever by Dr. Hale White. Dr. White discussed the diagnosis and treatment of typhoid fever, and gave some excellent practical advice on the subject of treatment.

#### University of Cambridge.

The Regius Professor of Physic at Cambridge, Dr. Clifford Allbutt, F.R.S., has been elected to a Fellowship at Gonville and Caius College. Dr. Allbutt graduated as a member of the College in 1859, and in 1860 obtained a first class in the Natural Sciences Tripos. He took the degrees of Bachelor of Medicine in 1861 and Doctor of Medicine in 1869.

#### University of Oxford.

Examinations in Medicine and Surgery, 1892.—The Regius Professor of Medicine gives notice that the Final Examination for the Degree of Bachelor of Medicine will commence on Monday, June 6th, at 10 a.m., in the Examination Schools. The Examination for the Degree of Master in Surgery will take place on Thursday, June 16th. The First Examination for the Degree of Bachelor of Medicine will commence on Thursday, June 23rd, at 10 a.m. The Secretary to the Board of Faculties gives notice that he will be in attendance at his office in the Clarendon Building on Saturday, May 21st, from 9.30 to 10.30 a.m., and from 2 to 3 p.m. for the purpose of receiving names of candidates for the Second Examination for the Degree of M.B.; on Friday, May 27th, from 2 to 3 p.m., for the purpose of receiving names of candidates for the Examination for the Degree of M.Ch.; and on Friday June 3rd, from 9.30 to 10.30 a.m., and from 2 to 3 p.m., for the purpose of receiving names of candidates for the First Examination for the Degree of M.B. Names with the statutable certificates and fees, may be sent to him by letter at any time not later than the above mentioned days respectively. No name can be entered for any of these Examinations unless the statutable certificates are exhibited, and the fees paid within the prescribed limit of time, namely, at least fourteen days before the first day of the week in which the examination is to be held.

#### Candidates for the Army Medical Service.

It is announced that in future every candidate for a commission in the Army Medical Staff will be required to possess, in addition to the qualifications previously laid down, a certificate of having acted as medical clinical clerk for six months, and as surgical dresser for another six months, not less than three months of each period being spent in hospital wards; also a certificate of having attended not less than three months' instruction at an ophthalmic hospital, whose course shall include instruction in the errors of refraction.

### PASS LISTS.

#### Victoria University.

FACULTY OF MEDICINE.

Second M.B. Examination.—The following have satisfied the examiners: C. S. Ashe, Owens College; W. J. Bowden, Owens College; S. W. Brook, Owens College; W. M. Brown, University College; A. Brushfield, Owens College; W. W. Clemesha, Owens College; T. S. Collin, Owens College; N. F. Edwards, Owens College; P. H. Fearnsides, Yorkshire College; F. W. Fish, Owens College; J. W. Hainsworth, Yorkshire College; J. P. Hall, Owens College; J. Howe, Owens College; R. T. Hughes, Owens College; A. W. Lilley, Owens College; T. U. Mercer, University College; E. Monks, Owens College; F. Radcliffe, Owens College; J. H. Ray, Owens College; J. F. Rimmer, Owens College; H. S. Smith, Owens College; W. L. Spink, Yorkshire College; O. B. Trumper, Yorkshire College; J. D. Whitaker, Owens College; P. Wilkinson, Owens College.

Final M.B. Examination (Part I).—The following have satisfied the examiners: R. Alcock, Owens College; J. C. Bawden, University College; H. C. Cadman, Owens College; J. B. Carter, Owens College; J. H. Crocker, Owens College; W. E. Davies, University College; A. Greenhalgh, Owens College; J. Healey, Owens College; C. R. Jones, University College; W. McClelland, University College; J. B. Mawdsley, University College; A. J. Partridge, Owens College; F. C. Scotson, Owens College; F. J. Woods, University College.

Final M.B. Examination (Part II).—The following have satisfied the examiners: A. E. Ash, Owens College; A. Bicknell, University College; J. W. Crawshaw, Owens College; A. J. Edwards, Owens College; H. J. Lightbody, University College; C. E. M. Lowe, Owens College; E. C. McCarthy, Owens College; R. W. Marsden, Owens College; C. B. Taylor, Owens College; J. H. Taylor, Owens College; R. T. Turner, Owens College.

The following have been awarded honours: First Class—J. W. Crawshaw, Owens College; J. H. Taylor, Owens College. Second Class—A. E. Ash, Owens College; E. C. McCarthy, Owens College; R. W. Marsden, Owens College; R. T. Turner, Owens College.

